# Family matters

**DOI:** 10.7554/eLife.43815

**Published:** 2018-12-28

**Authors:** Mark L Mayer, Timothy Jegla

**Affiliations:** 1 National Institute for Neurological Disorders and StrokeBethesdaUnited States; 2 Department of BiologyPennsylvania State UniversityUniversity ParkUnited States; 3 Huck Institute for the Life SciencesPennsylvania State UniversityUniversity ParkUnited States

**Keywords:** phylogenetics, ionotropic glutamate receptors, glutamate families, amphoxius, evolution, Other

## Abstract

Genome sequence data from a range of animal species are raising questions about the origins of glutamate receptors.

**Related research article** Ramos-Vicente D, Ji J, Gratacòs-Batlle E, Gou G, Reig-Viader R, LuísJ, Burguera D, Navas-Perez E, García-Fernández J, Fuentes-Prior P, Escriva H, Roher N, Soto D, Bayés À. 2018. Metazoan evolution of glutamate receptors reveals unreported phylogenetic groups and divergent lineage-specific events. *eLife* **7**:e35774 doi: 10.7554/eLife.35774

Receptors for the amino acid glutamate have a crucial role in the nervous system of nearly all animals. These proteins are split between two families: metabotropic glutamate receptors modulate the activity of neural networks, while ionotropic glutamate receptors mediate the transmission of signals between neurons. While we know a great deal about glutamate receptors, the gigabytes of data from recent genome sequencing projects provide a new opportunity to dissect how they have evolved.

These growing genomic data have also prompted new ideas about the evolution of animals. According to a new – and still controversial – version of the animal tree of life (Figure 1), comb jellies, or ctenophores, evolved first (Moroz et al., 2014; Ryan et al., 2013). Sponges (porifera) appeared next, followed by placozoans, jellyfishes (cnidarians) and, finally, bilaterians, vertebrates and invertebrates with bilateral body symmetry. If this revised tree is correct (Jékely et al., 2015; Ryan, 2014), neurons could have been lost twice during evolution because comb jellies have a nervous system but sponges and placozoans do not. Now, in eLife, Àlex Bayés of the Biomedical Research Institute Sant Pau in Barcelona and colleagues – including David Ramos-Vicente as first author – report new insights into the evolution of glutamate receptors by conducting a comprehensive study of these proteins across different animal groups (Ramos-Vicente et al., 2018).

**Figure 1.**
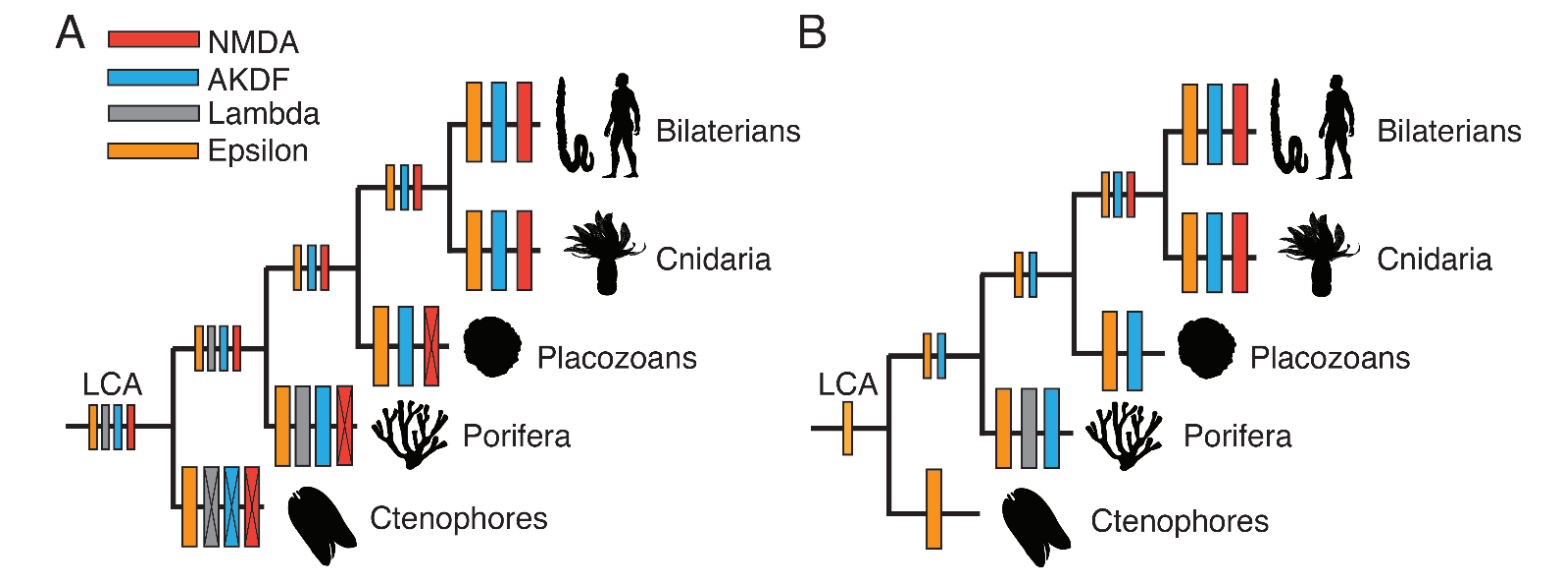
Two models for the evolution of glutamate receptors. (**A**) For many years, it was assumed that porifera (sponges) were the earliest animals, but some researchers now argue that instead, ctenophores (comb jellies) evolved first. Yet, the details of how important aspects of the nervous system evolved remain unclear. Ramos-Vicente et al. propose that the sub-families of ionotropic glutamate receptors (Epsilon: orange; Lambda: grey; AKDF: blue; NMDA: red) were present in the last common ancestor (LCA) of all animals, with certain sub-families being lost (indicated by a cross) one or more times during evolution. (**B**) An alternative scheme, which we favor, proposes that a precursor of Epsilon receptors was the only family present in the last common ancestor. Gene duplication would have led to the evolution of the AKDF sub-family in the ancestor of placozoans, cnidaria and bilaterians, with the Lambda sub-family appearing only in sponges. Finally, another gene duplication event would have given rise to NMDA receptors in cnidarians and bilaterians.

Image credit: Vignettes from phylopic.org. Ctenophores: Mali'o Kodis, photograph by Aqua-Photos; Porifera: Mali'o Kodis, photograph by Derek Keats (CC BY 3.0); Placozoans: Mali'o Kodis, photograph from Wikimedia (CC BY 3.0); Cnidaria: Qiang Ou (CC BY 3.0); Bilateria: Human (CC0), Hemichordata: Mali'o Kodis, drawing by Manvir Singh (CC BY 3.0).

Previously, researchers had identified four sub-families of ionotropic glutamate receptors, but they had mainly looked at vertebrate species. Now, Ramos-Vicente et al. muster data from other animal groups and propose a major reclassification that contains two new sub-families called Epsilon and Lambda. The NMDA receptors, which play a special role in vertebrates (Collingridge and Bliss, 1995), remain from the old classification, and the AKDF sub-family combines three other sub-families from the former model. Moreover, the team argues that all four sub-families were present in the last common ancestor of animals, with some being lost repeatedly during evolution (Figure 1A).

This would explain why the Epsilon sub-family is present in most animals today, whereas Lambda is only found in sponges. AKDF is carried by sponges, placozoans, jellyfish and bilaterians, but NMDA receptors exist only in these last two groups. However, we favor an alternative model in which the Epsilon sub-family evolved first, followed by the AKDF proteins. The Lambda receptors came next but only in sponges; then finally, the NMDA sub-family emerged in a common ancestor of jellyfish and bilaterians, persisting in these species (Figure 1B).

Amongst the newly identified sub-families, the Epsilon receptors are especially interesting because, like NMDA receptors, some of them are activated by glycine and others by glutamate. Such glycine receptors have previously been found in comb jellies (Alberstein et al., 2015; Yu et al., 2016), and now Ramos-Vicente et al. have identified them in a group of bilateral animals called lancelets, even though these organisms are separated from comb jellies by hundreds of millions of years of evolution. The receptors that get activated by glycine were more widespread than expected, with three receptors preferring glycine for every two favoring glutamate.

Deciphering the genetic sequence of a protein helps to predict its final structure. These analyses revealed that in comb jellies and lancelets, a subset of ionotropic glutamate receptors lacks the molecular features required to bind neurotransmitter amino acids, which suggests that they attach to other, as yet unidentified molecules. Surprisingly, when modeling the structure of one of the Epsilon receptors found in lancelets, it appeared that it might not be able to bind a ligand at all. This feature was seen in five Epsilon subunits and six AKDF subunits in these animals.

Many of the new receptors reported by Ramos-Vicente et al. have been identified only by analyzing their genetic sequences; their functional properties have yet to be studied either in native tissues, which is a challenge for many marine creatures, or by expressing these proteins in model organisms. When this is done, our understanding of the diversity of glutamate receptors will expand enormously.
